# Personal Communication Device Use by Nurses Providing In-Patient Care: Survey of Prevalence, Patterns, and Distraction Potential

**DOI:** 10.2196/humanfactors.5110

**Published:** 2017-04-13

**Authors:** Deborah L McBride, Sandra A LeVasseur

**Affiliations:** ^1^ Samuel Merritt University San Mateo, CA United States; ^2^ University of Hawaii at Manoa Hawaii State Center for Nursing School of Nursing and Dental Hygiene Manoa, HI United States

**Keywords:** distraction, mobile devices, nurses

## Abstract

**Background:**

Coincident with the proliferation of employer-provided mobile communication devices, personal communication devices, including basic and enhanced mobile phones (smartphones) and tablet computers that are owned by the user, have become ubiquitous among registered nurses working in hospitals. While there are numerous benefits of personal communication device use by nurses at work, little is known about the impact of these devices on in-patient care.

**Objective:**

Our aim was to examine how hospital-registered nurses use their personal communication devices while doing both work-related and non‒work-related activities and to assess the impact of these devices on in-patient care.

**Methods:**

A previously validated survey was emailed to 14,797 members of two national nursing organizations. Participants were asked about personal communication device use and their opinions about the impact of these devices on their own and their colleagues’ work.

**Results:**

Of the 1268 respondents (8.57% response rate), only 5.65% (70/1237) never used their personal communication device at work (excluding lunch and breaks). Respondents self-reported using their personal communication devices at work for work-related activities including checking or sending text messages or emails to health care team members (29.02%, 363/1251), as a calculator (25.34%, 316/1247), and to access work-related medical information (20.13%, 251/1247). Fewer nurses reported using their devices for non‒work-related activities including checking or sending text messages or emails to friends and family (18.75%, 235/1253), shopping (5.14%, 64/1244), or playing games (2.73%, 34/1249). A minority of respondents believe that their personal device use at work had a positive effect on their work including reducing stress (29.88%, 369/1235), benefiting patient care (28.74%, 357/1242), improving coordination of patient care among the health care team (25.34%, 315/1243), or increasing unit teamwork (17.70%, 220/1243). A majority (69.06%, 848/1228) of respondents believe that on average personal communication devices have a more negative than positive impact on patient care and 39.07% (481/1231) reported that personal communication devices were always or often a distraction while working. Respondents acknowledged their own device use negatively affected their work performance (7.56%, 94/1243), or caused them to miss important clinical information (3.83%, 47/1225) or make a medical error (0.90%, 11/1218). Respondents reported witnessing another nurse’s use of devices negatively affect their work performance (69.41%, 860/1239), or cause them to miss important clinical information (30.61%, 378/1235) or make a medical error (12.51%, 155/1239). Younger respondents reported greater device use while at work than older respondents and generally had more positive opinions about the impact of personal communication devices on their work.

**Conclusions:**

The majority of registered nurses believe that the use of personal communication devices on hospital units raises significant safety issues. The high rate of respondents who saw colleagues distracted by their devices compared to the rate who acknowledged their own distraction may be an indication that nurses are unaware of their own attention deficits while using their devices. There were clear generational differences in personal communication device use at work and opinions about the impact of these devices on patient care. Professional codes of conduct for personal communication device use by hospital nurses need to be developed that maximize the benefits of personal communication device use, while reducing the potential for distraction and adverse outcomes.

## Introduction

Personal communication devices (PCDs) such as basic and enhanced mobile phones (smartphones) and tablet computers that are owned by the user offer unprecedented convenience in our daily lives. Immediate social interaction and information retrieval have made PCDs indispensable for many individuals. Excluding employer-provided mobile communication devices, previous research has demonstrated that registered nurses who work in hospitals use their PCDs to access medical information, including drug and treatment information, as clinical decision tools, and to identify other clinical information that supports their ability to care for patients [1-3]. In addition to work-related PCD use, there is an ever-increasing number and diversity of recreational sites available to working nurses including video games, TV/movies, music, and social networking sites. Previous research reported that non‒work-related Internet use during working hours was increasingly common and that a majority of workers, regardless of age or occupational status, reported using PCDs to engage in non‒work-related activities while at work [4-6]. Notwithstanding the many advantages for clinicians and patients, little is known about the impact of PCDs on the work of clinicians. Katz-Sidlow et al [7] reported that 37% of medical residents and 12% of faculty self-reported using their smartphones to read or respond to personal emails or texts during in-patient attending rounds and that 15% of residents admitting using their smartphones to engage in other non-patient care uses during rounds. In addition, 19% of residents and 12% of attending physicians acknowledged missing important clinical information because of smartphone distraction during rounds and 34% of residents and 20% of attending physicians reported observing another team member miss important clinical information because of smartphone distraction during in-patient round attendance. Smith et al [8] surveyed surgical technicians about their use of their mobile phones while operating a heart-lung machine. He found that 55.6% self-reported using their mobile phone while working, 49.2% acknowledged sending text messages, 21% accessed personal email, 15.1% browsed the Internet, and 3.1% checked or posted on social networking sites. Although 92.7% of the respondents in Smith’s study reported that they had never been distracted by or had their performance at work negatively affected by their mobile phones and 98% reported that they had never made a medical error at work that could be attributed to their mobile phone use, 34.5% reported seeing another surgical technician distracted by their mobile phone during surgery. Safety concerns were reported by 78.3% of respondents who believed that mobile phones introduced a potentially significant safety risk to patients while working. These results suggest that while many clinicians were aware of the potential dangers of using PCDs while working, they may not be aware of their own decreased performance resulting from their PCD use.

Our study examined how registered nurses working on in-patient units used their PCDs at work (excluding lunch and breaks) and their opinions about how PCD use impacted their work and the work of their colleagues.

## Methods

In April 2014, 14,797 recruitment emails containing the link to a previously validated anonymous Web-based survey concerning personal communication device use at work were sent to members of the Academy of Medical Surgical Nurses (10,978 members) and the Society of Pediatric Nurses (3819 members). Two weeks after the initial email, a reminder email containing the survey link was sent to the membership. A total of 1268 respondents to the two emails met the inclusion criteria of having been employed as a registered nurse who averaged more than 20 hours a week of patient contact on an in-patient unit at some point within the last 5 years. These two national nursing organizations were selected because nurses often specialize in either adult or pediatric specialties and it was anticipated that there would be little overlap between the memberships of these two organizations.

The survey instrument was piloted in 2013 [9]. It consisted of four parts: (1) demographics, (2) PCD use at work, (3) opinions about PCD effects on registered nurses’ work, and (4) hospital policies concerning PCDs (Multimedia Appendix 1). Respondents were asked to rank statements concerning PCD use on a 5-point Likert scale to indicate their agreement. This scale was chosen because the piloted version demonstrated that it allowed for adequate response dispersion and meaningful PCD use identification among nurses. The survey pilot version was tested on hospital nurses for face validity, redundancy, and ease of use. Nurses were asked about their own PCD use, as well as their observations of other nurses’ use while working (excluding lunch and breaks). The statistical approach of this paper was to (1) describe the frequency of PCD use by nurses at work, (2) identify concerns and opinions among nurses regarding PCD use at work, and (3) compare the response of different demographic groups with regards to their use of PCDs and its effect on their work and the work of their colleagues. A chi-square test was conducted to examine whether the whole group of respondents preferred certain answer options to others and whether different groups of respondents present different opinions in the survey questions. A two-tailed *Z* test was used to examine the equality of proportions between each pair of respondent groups. For study purposes, a PCD was broadly defined as any basic mobile phone, enhanced mobile phone (smartphones), or tablet computer that was owned and paid for by the user. The definition of PCD excluded employer-provided mobile communication devices that were used for electronic medical information documentation or clinical communication among providers of any Health Insurance Portability and Accountability Act (HIPPA)‒protected patient information. Exempt status approval from the Institutional Research Board of the University of Hawai’i Human Subjects Committee was received on January 2, 2014 (CHS# 21816).

## Results

We received 1268 responses out of 14,797 potential participants (8.57%). Of the 14,797 potential participants, 58 were excluded because they did not have an email contact and 125 were excluded because they did not meet the inclusion criteria, primarily because they did not average more than 20 hours of patient contact per week on an in-patient unit. The average age of the respondents was 47.82 years, with 94.47% (1198/1268) of the respondents being female and 5.52% male (70/1268).

### Employment Characteristics

The majority of respondents were staff nurses (54.69%, 688/1258), while 14.39% (181/1258) were charge nurses, 10.65% (134/1258) nurse managers, 5.88% (74/1258) advanced practice nurses, 5.17% (65/1258) nurse faculty, 2.70% (34/1258) nurse executives, and 5.96% (75/1258) had other unidentified nursing-related positions.

### Respondents’ Use of Personal Communication Devices

Personal communication devices are pervasive in hospitals. Among respondents 98.67% (1142/1212) owned a PCD and 64.94% (804/1238) self-reported using their PCD often or always while at work (excluding lunch and breaks), 17.45% (216/1238) used their PCD sometimes while at work, and 17.61% (218/1238) rarely or never used their PCD while at work. Only 5.65% (70/1237) of respondents indicated that they never used a PCD while working. A chi-square test was conducted to examine whether these percentages statistically differ from the situation where respondents chose the answer options by chance alone (ie, one third for each option). The results showed significant difference, indicating that the distribution of use of PCD is not uniform (Χ^2^_2_=556.66, *P*<.001).

**Figure 1:**
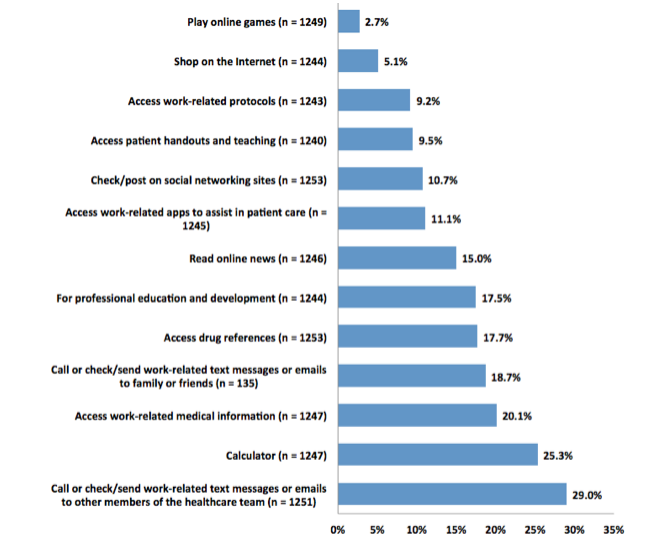
PCD use at work (excluding lunch or breaks). Although this figure is primarily descriptive, we performed t tests of each combination. Most of the variables are statistically significantly different from each other at the 5% level, and specifically only 6/78 combinations were not significant at the 5% level. These exceptions were access to drug references and professional education and development, access to patient handouts and access to work-related protocols, access to patient handouts and access to work-related apps, personal emails and access to drug references, personal emails and nursing or work-related information, personal emails and professional education and development.

Although it might be assumed that nurses would use their personal devices at work for only non‒work-related activities, respondents indicated that they frequently used their PCDs at work for activities that supported their work caring for patients. Both work-related and non‒work-related use of PCDs at work (excluding lunch and breaks) were assessed using 13 activities that were determined to be significant in the pilot study [9]. Work-related activities included checking or sending text messages or emails to other health care team members (29.02%, 363/1251), as a calculator (25.34%, 316/1247), accessing work-related medical information (20.16%, 251/1247), accessing drug references (17.48%, 219/1253), for professional education and development (17.52%, 218/1244), accessing work-related apps to assist in patient care (11.08%, 138/1245), accessing patient handouts and teaching material (9.52%, 118/1240), and accessing work-related protocols (9.17%, 114/1243). Non‒work-related activities included calling, checking or sending text messages or emails to family or friends (18.75%, 235/1253), reading online news (15.00%, 187/1246), checking or posting on social networking sites (6.98%, 87/1246), shopping (5.14%, 64/1244), and playing online games (2.72%, 34/1249). A *t* test was performed to compare work-related and non‒work-related activities. Work-related activities were found to be statistically significantly more likely than non‒work-related activities at the 5% significance level (*t*_2_=2.67, *P*<.001). This analysis showed that nurses were much more likely to use their PCDs at work for activities that supported their work caring for patients than for non‒work-related activities (Figure 1).

### Positive Impact on Work Performance

**Figure 2:**
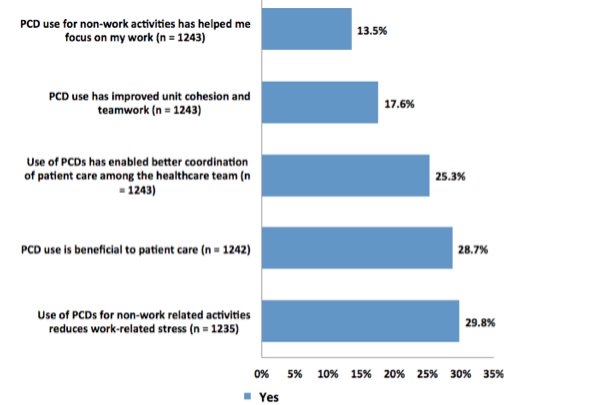
Experiences with PCD use positively affecting work performance. Although this figure is primarily descriptive, we performed t tests of each combination. All the means are statistically significantly different from each other at the 5% level of significance with the single exception of the mean for PCD use being beneficial to patient care and use of PCDs for non-work-related activities reduces work-related stress. The means of these two variables are not statistically different from each other.

Both positive and negative impacts of PCD use by hospital nurses were assessed using ten statements previously determined to be significant in the pilot study [9]. A minority of respondents agreed or strongly agreed that PCD use at work (excluding breaks and lunch) positively impacted their work including reduced stress (29.88%, 369/1235), was beneficial to patient care (28.74%, 357/1242), enabled better patient care coordination among the health care team (25.34%, 315/1243), improved patient safety (18.47%, 229/1240), improved unit cohesion and teamwork (17.70%, 220/1243), or improved one’s ability to focus on work (13.52%, 168/1243) (Figure 2). Although it seems intuitive that non‒work-related PCD use at work would negatively affect productivity and performance by taking away time from work-related activities, these results indicate that some nurses believe that use of PCDs at work has benefits both for the individual and for the organization as a whole. An unanswered question involves whether these reported benefits could potentially violate HIPPA laws related to the transmission of protected patient information on unsecured networks.

### Negative Impact on Work Performance

**Figure 3:**
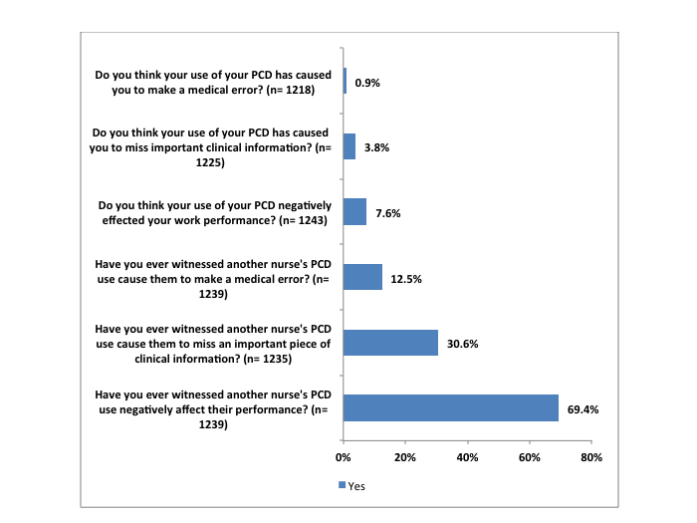
Experiences with PCD use negatively affecting work performance.

Three survey questions assessed self-reported and witnessed performance decrements associated with PCD use in the following areas: (1) negative performance, (2) medical errors, and (3) missed clinical information (Figure 3). For the purposes of this survey, a medical error was defined as an adverse effect on care, including a near miss or sentinel event.

Figure 3 presents three pairs of survey question results, with each pair involving the experiences with PCD use negatively affecting respondents’ own work performance as well as their observations of PCD use negatively affecting other nurses’ work performance. While presenting each pair of results, we also conducted *Z* tests on the equality of proportions and present the results in parentheses. Respondents were more likely to report that PCD use had negatively affected another nurse’s work performance than their own (*Z*=31.67, *P*<.001). A majority (69.41%, 860/1239) of respondents had witnessed other nurses’ PCD use negatively affect their work performance. In contrast, few respondents (7.56%, 94/1243) acknowledged that their own PCD use had negatively affected their work performance.

In addition, less than one percent (0.90%, 11/1218) of respondents reported having made a medical error because of PCD distraction while 12.5% (155/1239) reported having witnessed a colleague make a medical error because of their PCD use (*Z*=-11.46, *P*<.001). Similarly, 3.84% (47/1225) reported having missed an important piece of clinical information because of PCD distraction, compared to 30.6% (378/1235) who reported witnessing a colleague miss important clinical information because of their PCD use (*Z*=-17.56, *P*<.001). The significant results indicate that respondents were ten times more likely to report witnessing PCDs negatively affecting the work of their colleagues than to report PCD use negatively affecting their own work.

### Overall Effects of Personal Communication Device Use While Working

Over two-thirds (69.06%, 848/1228) of respondents believed that PCD use by nurses in hospitals had more negative than positive effects on patient care, whereas less than a third (30.94%, 380/1228) of respondents believed that PCD use had more positive than negative effects on patient care. Chi-square test results indicated that significantly more respondents considered PCD use as having more negative than positive effects on patient care (Χ^2^_2_=178.36, *P*<.001).

**Figure 4:**
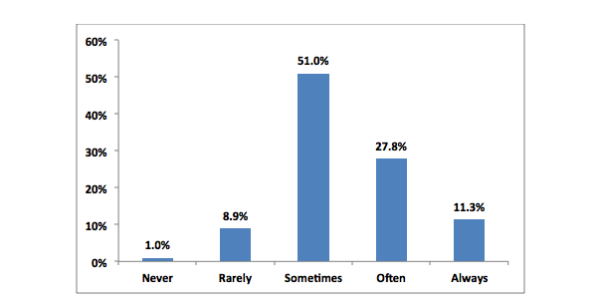
Belief about whether PCDs are a serious distraction at work.

In addition, 39.07% (481/1231) of respondents reported that PCDs were always or often a distraction while working, 51.02% (628/1231) reported that PCDs were sometimes a distraction, and 9.91% (122/1231) felt that PCDs were rarely or never a distraction (Figure 4).

### Age and Personal Communication Device Use While at Work

**Figure 5:**
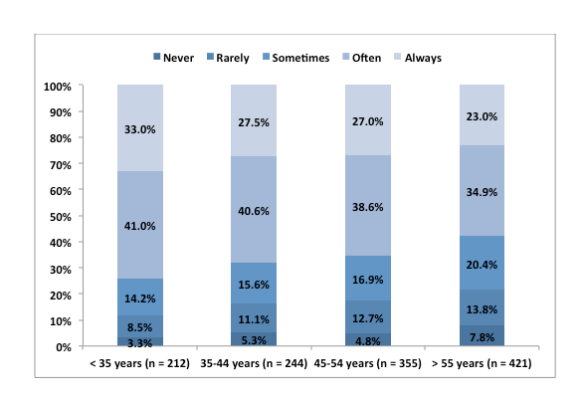
PCD use at work by age.

The chi-square test was used to determine how age affected the frequency of use of PCDs at work. When comparing opinions across different respondent segments, the clearest trend and biggest differences existed across the youngest and oldest age groups. Younger respondents reported greater PCD use while at work than older respondents, and chi-square test results indicated that such a difference was statistically significant (Χ^2^_2_=17.85, *P*<.001). Three quarters (74.1%, 157/212) of respondents younger than 35 years old reported using a PCD often or always while at work. This percentage diminished across older age groups until, among those 55 or older, 58.0% (244/421) reported using a PCD often or always while at work (Figure 5).

Younger respondents were more likely to use their PCD for work-related activities than older respondents (Table 1). Specifically, 37.9% (80/211) of respondents under age 35, compared to 15.7% (67/428) of those over age 54 reported using their PCD as a calculator often or always (*Z*=6.29, *P*<.001). Similar differences existed for using a PCD to access drug references in younger nurses (25.9%, 55/212) versus older (15.1%, 65/430) (*Z*=3.31, *P*<.001).

Younger respondents reported believing that PCD use was beneficial to patient care at higher rates than older respondents (Table 2). For example, just over half of respondents under 35 years of age (51.9%, 110/212) agreed or strongly agreed that PCD use reduced stress, compared to a fifth of respondents older than 54 years of age (21.2%, 90/424) who agreed or strongly agreed that this was true (*Z*=7.85, *P*<.001).

**Table 1:** PCD activities at work by age^a^.

PCD activities	<35 years (N=211-212) n (%)	35-44 years (N=242-246) n (%)	45-54 years (N=354-360) n (%)	>54 years (N=424-431) n (%)
Call or check/send work-related text messages or emails to other members of the health care team	67 (31.8)	76 (30.9)	101 (28.2)	117 (27.2)
Calculator	80 (37.9)^c,d^	78 (32.2)^c,d^	88 (24.4)^d^	67 (15.7)
Access work-related medical information	54 (25.6)	44 (18)	68 (18.9)	84 (19.7)
Call or check/send text messages or emails to family or friends	50 (23.7)^c,d^	63 (25.6)^c,d^	56 (15.6)	65 (15.1)
Access drug references	55 (25.9)^b,c,d^	45 (18.3)	58 (16.2)	65 (15.1)
For professional education and development	33 (15.6)	43 (17.6)	62 (17.3)	77 (18.1)
Read online news	33 (15.6)	44 (18.1)	50 (14)	60 (14)
Access work-related apps to assist in patient care	32 (15.2)^c^	29 (11.9)	34 (9.5)	44 (10.3)
Access patient handouts and teaching	20 (9.4)	20 (8.2)	35 (9.9)	44 (10.4)
Access work-related protocols	15 (7.1)	25 (10.2)	32 (9)	43 (10.1)
Check/post on social networking sites	27 (12.7)^c,d^	22 (9.1)^d^	25 (7)^d^	13 (3)
Shop on the Internet	12 (5.7)	16 (6.6)	19 (5.3)	17 (4)
Play online games	9 (4.2)	5 (2)	11 (3.1)	9 (2.1)

^a^We conducted equality of proportion tests to examine whether each pair of age groups are significantly different in the percentages reporting the activities above often or always. The significant differences based on the statistical test results are also presented in this table. The cells with superscripts indicate that the corresponding group has a significantly larger proportion of respondents reporting the corresponding activity often or always compared to each group in the superscript at 5% level of significance.

^b^35-44 years group.

^c^45-54 years group.

^d^>54 years group.

**Table 2:** Agreement level with statements about PCD work use by age^a^.

Statements about PCD use	<35 years (N=210-212) n (%)	35-44 years (N=240-244) n (%)	45-54 years (N=353-356) n (%)	>54 years (N=424-429) n (%)
Use of PCDs at work reduces stress.	110 (51.9)^b,c,d^	83 (34.6)^c,d^	84 (23.8)	90 (21.2)
PCD use is beneficial to patient care.	76 (36.2)^c,d^	76 (31.1)	90 (25.4)	114 (26.6)
Use of PCDs has enabled better coordination of patient care among the health care team.	73 (34.6)^b,c,d^	58 (23.8)	85 (24.1)	99 (23.1)
PCD use has improved unit cohesion and teamwork.	52 (24.5)^b,c,d^	41 (16.9)	53 (14.9)	74 (17.4)
PCD use helps me focus on my work.	30 (14.3)	36 (14.8)	57 (16)^c^	44 (10.3)

^a^We conducted equality of proportion tests to examine whether each pair of age groups are significantly different in the percentages (strongly) agreeing with the statements above. The significant differences based on the statistical test results are also presented in this table. The cells with superscripts indicate that the corresponding group has a significantly larger proportion of respondents (strongly) agreeing with the corresponding statement compared to each group in the superscript at 5% level of significance.

^b^35-44 years group.

^c^45-54 years group.

^d^>54 years group.

Respondents under age 25 were the only age group in which more than half (56%, 10/18) believed PCD use had a more positive than negative effect on patient care, though such proportion might be due to chance as the chi-square test is not statistically significant (Χ^2^_2_=0.22, *P*=.637). For all other age groups, more than half reported that PCD use had a more negative than positive effect on patient care. This percentage increased across age groups, up to the age of 65 years or older where over three-quarters (77%, 23/30) believed that PCDs had a more negative than positive effect on patient care (Χ^2^_2_=8.53, *P*=.003).

Older respondents were more likely than younger respondents to believe PCD use was a distraction at work. Over half of respondents over age 65 (57%, 17/30) believed PCD use was always or often a serious distraction at work, compared to just over a quarter of respondents between age 25 and 34 years (27.6%, 53/192). Equality of proportion test results indicate that such a difference is statistically significant (*Z*=3.19, *P*=.001).

## Discussion

### Principal Findings

Nurses and their patients benefit from the many capabilities of personal communication devices on in-patient units. PCDs contain medical references, facilitate communication transfer, and assist in patient care coordination on in-patient units. However, despite their significant advantages, PCDs introduce another source of distraction into the hospital environment. While some interruptions can be beneficial, others, even those that are self-initiated, can be distracting and detrimental to patient care. Studies from psychology and education have reported on the negative consequences of distraction on task performance. Mobile phone use while operating a motor vehicle can be hazardous. Lesch and Hancock [10] reported on the awareness of motor vehicle drivers of their reduced driving ability while operating a mobile phone. They found that drivers were oblivious to their reduced driving ability caused by concurrent mobile phone use and that there was a great discrepancy between driver perceptions and actual driving performance. Strayer et al [11] found that drivers described other drivers using their mobile phones as driving poorly but reported that their own driving during mobile phone use remained normal, even when the results of driving performance tests showed otherwise. These results concur with this study’s results: that there is an apparent disconnect between self-reported and observed performance among respondents about PCD use. Although respondents self-reported low levels of performance decrements, the significantly higher level of reported witnessed performance decrements should be cause for concern because it raises the possibility of patient safety issues.

Although PCD use at work differs from other types of potentially nonsanctioned behaviors, some insight may be gained by looking at research into another form of rule breaking, academic cheating. Jorden [12] reported that student cheaters differed from noncheaters in a number of different ways including their perceptions of social norms regarding cheating, their knowledge of institutional policy regarding cheating, and their attitudes toward cheating. According to Jordan, lack of knowledge of institutional policy was the best predictor of student cheating, followed by positive attitudes about social norms about cheating. A 2014 survey of US hospitals [13] found that 88% of US hospitals reported having a policy on PCD use by nurses at work. The lack of knowledge of a PCD policy at work and perceptions of peer comparisons and social acceptability of PCD use at work (eg, “Everyone else is doing it”), which may or may not be accurate, influence attitudes and behaviors at work. Complex interactions of many variables likely contribute to the risks of continuing PCD use in the face of performance decrements by nurses.

Unlike nurse demographics—which offer little guidance to institutions for curbing misuse of PCDs—attitudes, knowledge of PCD policies, and social comparison factors are potentially open to manipulation. For example, persuasive ethical arguments for restricted use of PCDs on nursing units may be addressed in workforce training, including during hospital orientation and unit training programs. This training could contain information about institutional policies and address issues of professionalism and peer accountability. These types of programs may reinforce and increase the attitudes towards responsible PCD use that many nurses hold and may dissuade them from engaging in high-risk PCD use.

### Limitations

Several limitations of this study should be acknowledged. Self-selection bias affects any survey that allows respondents to decide whether to participate. To mitigate this potential problem, the characteristics of the respondents in our study were compared with those of California-based registered nurses. The respondents were not systematically different from those of the state’s average registered nurse in terms of gender, age, race/ethnicity, job title, and experience with PCDs.

Another weakness of the study was the low response rate. Because measuring the relation between nonresponse and the accuracy of a survey statistic is complex and expensive, few rigorously designed studies provide empirical evidence to document the consequences of lower response rates. However, Holbrook et al [13] examined the results of 81 national surveys with response rates varying from 5% to 54%. They found that surveys with much lower response rates were only minimally less accurate than those with higher response rates. Nevertheless, the low response rate did increase the statistical error in the analysis and prevent extensive subanalyses. Further testing with a higher response rate would be necessary to overcome this limitation.

The self-reported nature of the data increases the risk of response bias as respondents may overreport or underreport their use of PCDs at work in order to present themselves in a socially desirable manner. Previous research has shown that study participants demonstrate lower social desirability when they respond to an online survey compared to a paper questionnaire [14]. This survey focuses on making medical errors and missing important clinical information, which could reflect badly on study participants. Therefore, we used an online survey to ensure that the impact of social desirability was kept to a minimum and anonymity was protected. As a result of these issues, data comparisons should be interpreted with caution.

### Conclusion

A majority of nurses in our study agreed that PCD use can be a significant distraction while providing in-patient care. Although many hospitals have policies outlining appropriate PCD use by clinicians at work, frequently hospitals allow workers to decide on their own how and when to use their devices. This presumes that workers can accurately assess the risks associated with PCD use and can appropriately modify their behavior. The results of this study suggest that nurses expressed a disproportionately high confidence in their ability to manage the risk associated with PCD use at work and may not be able to accurately assess when it is appropriate to use their PCDs or to modify their behavior accordingly. The development and implementation of professional codes of conduct for PCD use on in-patient units are important for patient safety. Guidelines on PCD use should be developed that maximize the benefits of PCD use in the hospital environment, while reducing the potential for distraction and adverse outcomes.

## Appendix

Multimedia Appendix 1 Nurses' use of personal communication devices questionnaire.

## Abbreviations

HIPPA: Health Insurance Portability and Accountability Act
PCD: personal communication device
