# Supplementary material for: Personal Communication Device Use by Nurses Providing In-Patient Care: Survey of Prevalence, Patterns, and Distraction Potential
Source: JMIR Hum Factors. 2017 Apr 13;4(2):e10. doi: 10.2196/humanfactors.5110 (PMC5408133; doi:10.2196/humanfactors.5110)
Supplement: Multimedia Appendix 1 [file humanfactors_v4i2e10_app1.pdf]

Thank you for taking the time to fill out this survey. Your responses will help improve the current medical policy and practice, and reduce potential medical errors to patients.

1) What is your gender?

- ☐ Male
- ☐ Female

2) How old are you? (pull down menu)

- ☐ 19
- ☐ 20
- ☐ 21
- ☐ 22
- ☐ 23
- ☐ 24
- ☐ 25
- ☐ 26
- ☐ 27
- ☐ 28
- ☐ 29
- ☐ 30
- ☐ 31
- ☐ 32
- ☐ 33
- ☐ 34
- ☐ 35
- ☐ 36
- ☐ 37
- ☐ 38
- ☐ 39
- ☐ 40
- ☐ 41
- ☐ 42
- ☐ 43
- ☐ 44
- ☐ 45
- ☐ 46
- ☐ 47
- ☐ 48
- ☐ 49
- ☐ 50
- ☐ 51
- ☐ 52
- ☐ 53
- ☐ 54
- ☐ 55
- ☐ 56
- ☐ 57
- ☐ 58

☐ 59
☐ 60
☐ 61
☐ 62
☐ 63
☐ 64
☐ 65
☐ 66
☐ 67
☐ 68
☐ 69
☐ 70
☐ 71
☐ 72
☐ 73
☐ 74
☐ > 75

3) What is your race/ethnicity? (check one)
☐ American Indian or Alaska Native
☐ Asian
☐ Black/African American
☐ Native Hawaiian or other Pacific Islander
☐ White/Caucasian
☐ Hispanic/Latino
☐ Other

4. Have you been employed as a registered nurse in a medical facility sometime during the last 5 years?
☐ No
☐ Yes

5. How would you characterize the location of your primary place of employment?
☐ Inner city
☐ Rural
☐ Urban
☐ Suburban
☐ Other

6. Please identify the position title that most closely corresponds to your nursing practice position.
☐ Consultant
☐ Nurse Researcher
☐ Nurse Executive
☐ Nurse Faculty
☐ Nurse Manager
☐ Advanced Practice Nurse
☐ Staff Nurse
☐ Charge Nurse
☐ Other-Health Related
☐ Other-Not Health Related

7. Please select the major activity that best describes your role in your primary nursing position.

☐ Not Applicable
☐ Patient Care and Education
☐ Student/Staff-Teaching/Training/Instruction
☐ Administration/Management
☐ Quality Assurance
☐ Case Management
☐ Research
☐ Other

8. On average, how many hours per week do you have patient contact in an in-patient setting?

☐ 0-5 hours per week
☐ 6-10 hours per week
☐ 11-15 hours per week
☐ 16-20 hours per week
☐ > 20 hours per week

9. How long have you worked as a registered nurse in a medical facility?

☐ Less than 1 year
☐ 1 to 3 years
☐ More than 3 years but less than 5 years
☐ 5 or more years but less than 10 years
☐ 10 years or more
☐ Not applicable

10. What U.S. state are you currently employed in as a registered nurse?
(drop down menu of states and DC)

11. How would you characterize your primary place of employment?

☐ Not-for-profit
☐ For-profit
☐ State or local government community hospital

12. How many beds does your primary place of employment have?

☐ 6-24 beds
☐ 25-49 beds
☐ 50-99 beds
☐ 100-199 beds
☐ 200-299 beds
☐ 300-399 beds
☐ 400-499 beds
☐ 500 beds or more
☐ Other

13. In general, how do you feel about personal communication devices (cell phone, smartphone or tablet computer)?

☐ Strongly negative
☐ Slightly negative

□ Neutral
□ Slightly positive
□ Strongly positive

14. How often do you use your personal communication device (cell phone, smartphone or tablet computer) while at work (excluding lunch and breaks)?

□ Never
□ Rarely
□ Sometimes
□ Often
□ Always

15. What best describes your primary personal communication device?

□ I don't have a personal communication device.
□ I have a basic personal communication device (cell phone only).
□ I have a personal communication device (cell phone and texting).
□ I have a smart phone (cell phone, texting, email, Internet access, apps).
□ I have a tablet computer.

16. On an average workday describe your use of your personal communication while at work (excluding breaks and meal times).

	Never	Rarely	Sometimes	Often	Always
I access work drug references.					
I access work-related nursing/medical information.					
I use the device as a calculator for nursing/medical formulas.					
I access work-related protocols.					
I access work-related apps that assist my patient care.					
I access sites for professional education and development.					
I access sites for patient handouts and teaching.					
I call or check/send work related text messages or emails to other members of the healthcare team.					
I read online news					
I call or check/send text messages or emails to family or friends.					
I shop on the internet.					
I check/post on social networking sites.					
I play online games.					

17. Please rate how your feel about the following statements about the use of personal communication devices while working (excluding breaks or meal times).

	Strongly disagree	Disagree	Neutral	Agree	Strongly agree
The use of my personal communication device for non-work related activities has distracted me while working.					
The use of my personal communication device for non-work related activities has negatively effected my performance while working.					
I have witnessed another nurse whose personal communication device use was negatively effecting his/her performance while working.					
The use of my personal communication device for non-work related activities has helped me focus on my work.					
The use of my personal communication devices has enabled better coordination of patient care among the healthcare team.					
Personal communication device use has improved unit cohesion and teamwork.					
Personal communication device use has improved patient safety.					
Patient communication device use is beneficial to patient care.					
Personal communication device use at work for non-work related activities improves my ability to focus on my work.					
Use of personal communication devices at work for non-work related activities reduces work-related stress.					

18. Has the use of a personal communication device ever negatively effected your performance as a nurse?

- No
- Yes

19. Have you ever witnessed another nurse colleague’s personal communication device use negatively effect their performance?

☐ No
☐ Yes

20. Have you ever made a medical error (defined as an adverse effect of care, including a near miss or a sentinel event) because you were distracted by the use of your personal communication device?

☐ No
☐ Yes

21. Have you ever witnessed a nurse colleague make a medical error (defined as an adverse effect of care, including a near miss or sentinel event) because they were distracted by cell phone/texting?

☐ No
☐ Yes

22. Do you think you have ever missed an important piece of clinical information because you were distracted by the use of your personal communication device?

☐ No
☐ Yes

23. Have you ever witnessed a colleague miss an important piece of clinical information because they were distracted by their personal communication device while working?

☐ No
☐ Yes

24. Do you think that personal communication devices can be a serious distraction during work?

☐ Never
☐ Rarely
☐ Sometimes
☐ Often
☐ Always

25. On balance, do you think the use of personal communication device use by nurses on the unit has a more positive or negative effect on patient care?

☐ More Negative
☐ More Positive

26. If multitasking is consuming more than one stream of media content at the same time, how would you characterize yourself?

☐ Heavy multitasker
☐ Average multitasker
☐ Light multitasker
☐ I never multitask

27. Does your employer have a policy on the use of personal communication devices at work?

☐ No
☐ Yes

28. Do you think your employer should establish a policy for personal communication device use at work?

- No Applicable, there is a policy in place
- No
- Yes

29. What best describes your opinion of how nurses should use their personal communication devices should at work (excluding breaks and meal times):

- Personal communication devices should never be used while working.
- Personal communication devices should only be used at work for work-related activities.
- Personal communication devices should only be used at work for urgent personal reasons.
- Personal communication device use at work for any reason is fine, as long as one uses common sense and good judgment.

30. Please select the column that best describes your opinion about the use of personal communication devices at work:

	Strongly negative	Slightly negative	Neutral	Slightly positive	Strongly positive
How do you feel about a nurse when you see them using their personal communication device on the unit?					
How do you think patients feel when they see a nurse using their personal communication device on the unit?					
How do you think other healthcare staff feel when they see a nurse using their personal communication device on the unit?					

Thank you for taking the time to fill out this survey.
